# Brain network clustering with information flow motifs

**DOI:** 10.1007/s41109-017-0046-z

**Published:** 2017-08-03

**Authors:** Marcus Märtens, Jil Meier, Arjan Hillebrand, Prejaas Tewarie, Piet Van Mieghem

**Affiliations:** 1 Delft University of Technology, Faculty of Electrical Engineering, Mathematics and Computer Science, P.O Box 5031, Delft, The Netherlands; 2 Department of Clinical Neurophysiology and Magnetoencephalography Center, Neuroscience Campus Amsterdam, VU University Medical Center, Amsterdam, The Netherlands; 3 Sir Peter Mansfield Imaging Centre, School of Physics and Astronomy, University of Nottingham, Nottingham, UK

**Keywords:** Network motifs, Network clustering, Brain networks, Information flow, Effective connectivity

## Abstract

Recent work has revealed frequency-dependent global patterns of information flow by a network analysis of magnetoencephalography data of the human brain. However, it is unknown which properties on a small subgraph-scale of those functional brain networks are dominant at different frequencies bands. Motifs are the building blocks of networks on this level and have previously been identified as important features for healthy and abnormal brain function. In this study, we present a network construction that enables us to search and analyze motifs in different frequency bands. We give evidence that the bi-directional two-hop path is the most important motif for the information flow in functional brain networks. A clustering based on this motif exposes a spatially coherent yet frequency-dependent sub-division between the posterior, occipital and frontal brain regions.

## Introduction

The application of network science to neuroscience has provided a new research perspective on the organization of brain networks from healthy subjects and patients suffering from neurological disorders (Stam and Van Straaten 2012; Bullmore and Sporns 2009). A recent study by Hillebrand et al. (2016) observed frequencydependent global patterns of information flow based on magnetoencephalography (MEG) data of healthy subjects. However, little is known about the underlying mesoscale level in terms of network motifs at which these flows occur.

To analyze information flow, the pairwise measure of transfer entropy (TE) has often been applied (Schreiber 2000). For a pair of time series *X* and *Y*, TE quantifies the improvement in predicting the future of *X* when considering both the current value of *X* and the current value of *Y*, compared to only using the current value of *X*. At the level of brain regions, the TE value is classified as a measure of effective connectivity between two regions.

Recently, an extension of the TE that is based on phase information (Rosenblum et al. 2001), the Phase Transfer Entropy (PTE), has been proposed in order to lower the computational costs and complexity (Lobier et al. 2014; Paluš M and Stefanovska 2003). After calculating all pairwise PTE values, functional brain networks with nodes representing brain regions and link weights inheriting their pairwise effective connectivities, can be constructed so that the topology of these networks can be characterized.

Based on the pairwise PTE values, Hillebrand et al. (2016) observed that for higher frequency bands, *alpha1*, *alpha2* and *beta*, the global information flow was predominantly from posterior to anterior brain regions, whereas the pattern was opposite for the low frequency *theta* band. The latter, an anterior-to-posterior pattern, was also discovered in electroencephalography (EEG) data (Dauwan et al. 2016). It was hypothesized that the information flow in resting-state networks is likely driven by the strong posterior structural hubs and their high levels of neuronal activity (Hillebrand et al. 2016; Moon et al. 2015; Tewarie et al. 2014). However, the opposite directions of information flow are not yet fully understood.

Another biological explanation for the reverse patterns could be the Default Mode Network (DMN), which is the network of brain regions that are active during resting-state. The DMN consists of two interacting subsystems: the temporal system, which is responsible for memory, and the fronto-parietal system, which is essential for self-relevant mental simulations (Buckner et al. 2008). These two subsystems seem to exist in parallel, though at different frequencies, and their interaction represents an integration mechanism for brain functions (Edelman and Gally 2013). This hypothesis is strengthened by results from invasive animal recordings of the visual cortex (Van Kerkoerle et al. 2014; Bastos et al. 2015), where the opposite directions of information flow have been connected with the process of memory consolidation (Sirota et al. 2008).

In this study we investigate the information flow patterns with regard to a smaller scale for different frequency bands. On the mesoscale level of brain networks, network motifs have been identified as a valuable feature by many previous studies (Sporns and Kötter 2004; Honey et al. 2007; Sporns et al. 2007). Motifs are frequently occurring subgraphs of networks, typically consisting of three or four nodes (Milo et al. 2002). Previous studies were able to link structural and functional brain networks with regard to their motifs to describe flexibility in switching between different brain functions (Battaglia et al. 2012) and for coupling of brain dynamics (Battiston et al. 2017). Furthermore, changes in the motif frequencies of so-called progression networks for patients suffering from Alzheimer’s disease have been discovered (Friedman et al. 2015), showing that motif analysis may provide potentially powerful new biomarkers.

The importance of motifs has not only been studied for brain networks, but also for various others, like biological transcription networks (Mangan and Alon 2003), food webs (Kondoh 2008) or transportation and mobility networks (Schneider et al. 2013). In order to link motifs to the modular organization often present in such networks, Benson et al. (2016) proposed a new algorithm for motif-based clustering. Since this algorithm identified clusters of functional importance in the neuronal network of the *C. Elegans*, it appears to be a promising approach to analyze the higher-order organization of human brain networks. Our previous study (Meier et al. 2016) gave evidence that clusters obtained by this algorithm are indeed meaningful for effective connectivity networks constructed using a similar metric, the directed phase transfer entropy (dPTE). Here, we extend this preliminary work with results for PTE (as compared to dPTE) and for two frequency bands, the *alpha2* and the *theta* band.

## From measurements to directed networks

### Measuring information flow in the brain

**Fig. 1:**
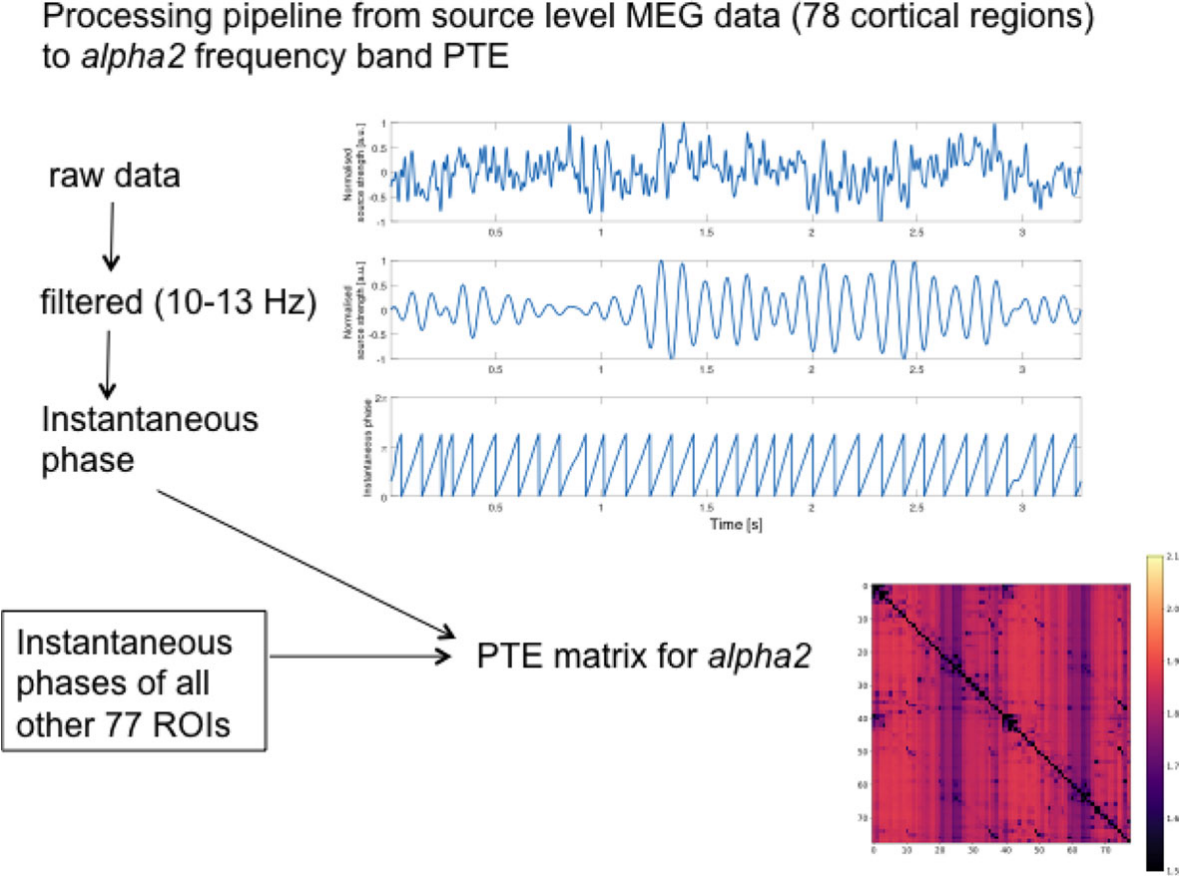
Processing pipeline from source level MEG data (78 cortical regions) to *alpha2* frequency band PTE matrix. The figure shows an example time series of a single ROI for a single epoch. In order to calculate the PTE matrices, we need the instantaneous phases of all 78 cortical regions. The PTE matrix entry (*i*,*j*) corresponds to the PTE value from region *i* to *j*

MEG measures the magnetic field fluctuations induced by neuronal activity (Hämäläinen et al. 1993). The data for our analysis is based on MEG recordings in 67 healthy subjects from a preceding study (Tewarie et al. 2014) and was used to show the frequency-dependence of the global information flow in the brain. In particular, it was shown that the *alpha2* band at 10-13 Hz has a strong back to front information flow, while the *theta* band at 4-8 Hz has a strong front to back information flow (Hillebrand et al. 2016). This current study is based on the *alpha2* and *theta* band as well. Figure 1 gives a schematic overview of our processing pipeline, from an example time series of source level MEG data towards obtaining the PTE matrices for the *alpha2* frequency band (*theta* frequency band data follows a similar processing).

**Fig. 2:**
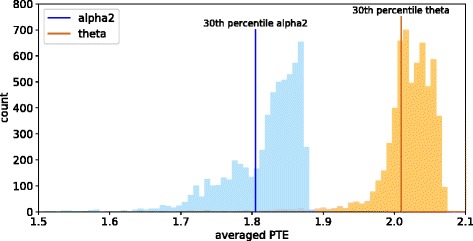
PTE between each possible pair of ROIs averaged over all subjects and measurement epochs. In total, 6006 average PTEs are displayed as a histogram with 100 bins for each of the two frequency bands. The *alpha2* frequency band (shown in *blue*) has on average lower PTEs than the *theta* frequency band (shown in *orange*). The *vertical lines* mark the 30th-percentile of each distribution

From the MEG measurements, we obtained^1^ phase time series (Rosenblum et al. 2001) from 78 different cortical regions of interest (ROIs) based on the Hilbert transform. We denote a possible value of the instantaneous phase of the signal of region *X* at time *t* by *x*_*t*_ and abbreviate the probability that the phase of *X* equals *x*_*t*_ at an arbitrary time point *t* to Pr[*X*_*t*_=*x*_*t*_]=Pr[*x*_*t*_]. The information flow between two ROIs, *X* and *Y*, is then quantified by the Phase Transfer Entropy (Lobier et al. 2014)

1 $$\begin{array}{@{}rcl@{}} \text{PTE}_{XY}(h)= \sum \text{Pr} \left[ x_{t+h}, x_{t}, y_{t} \right] \times \log \left(\frac{\text{Pr} \left[ x_{t+h} | x_{t}, y_{t} \right] }{\text{Pr} \left[ x_{t+h} | x_{t} \right]} \right),  \end{array} $$

for a certain time delay *h*, where the sum runs over all possible values *x*_*t*_, *x*_*t*+*h*_ and *y*_*t*_ of the instantaneous phases of the signals. The (joint) probabilities are determined over histograms of their occurrences in an epoch (Lobier et al. 2014). Following Hillebrand et al. (2016), we fix *h* at

2 $$ h = \frac{N_{s} \cdot N_{ROI}}{N_{\pm}},  $$

where *N*_*s*_=4096 and *N*_*ROI*_=78 are the number of samples in an epoch and the number of ROIs, respectively, and *N*_±_ counts the number of sign changes for the phase across time and ROIs. For clarity, *h* will be omitted from the notation and we use only PTE_*XY*_ instead of PTE_*XY*_(*h*) in the remainder. It should be noted, that the PTE of two regions *X* and *Y* is asymmetric, so PTE_*XY*_=PTE_*YX*_ does not hold in general. In order to remove individual bias of the measurements, all pairwise PTE values are averaged over all subjects and all epochs. A histogram of those averaged PTEs is shown in Fig. 2 for the *alpha2* and *theta* band.

### Network construction

The pairwise PTE values between all 78 ROIs imply a fully connected network *G*_PTE_ where each ROI is a node and the PTE is the weight of each link. In order to filter out noise and focus on the most important connections possessing the highest PTE values, all links with a PTE below or equal a certain threshold *τ* are discarded (set to zero) and all links above *τ* remain without a weight (set to one). This procedure eliminates weak connections which might otherwise obscure the inherent topology induced by significantly stronger connections. If (for a fixed *h*) PTE_*XY*_>*τ* and PTE_*YX*_>*τ* for two ROIs *X* and *Y*, a bi-directional link between *X* and *Y* is set. Similarly, for PTE_*XY*_>*τ*≥PTE_*YX*_, only a uni-directional link from *X* to *Y* is set. Thus, by selecting an appropriate threshold *τ*, the fully connected weighted network *G*_PTE_ is transformed into a sparser, directed and unweighted network *G*(*τ*), also known as binary directed network.

Finding an appropriate threshold *τ* is a challenge in itself (van Wijk et al. 2010), which we will not undertake, since one singular value for *τ* will not be needed in our approach here. Instead, we consider a class of networks *G*(*τ*) created by sampling *τ* from an interval [*τ*_*min*_,*τ*_*max*_]. Setting *τ*=0 results in a fully connected network whereas setting *τ* to the maximum of all PTE values results in an empty network of 78 isolated nodes. Clearly, these extreme thresholds provide networks that lack structure and present no insight. To avoid constructing such degenerate networks, we pick a narrower interval as follows:

We set *τ*_*max*_ to be the smallest threshold at which the obtained network is still weakly connected, i.e. has no isolated nodes. To avoid too many weak connections, *τ*_*min*_ is set to the 30th-percentile of the PTE distributions (see Fig. 2). This value eliminates a fair amount of weak connections while the majority of the strongest connections persist.

**Fig. 3:**
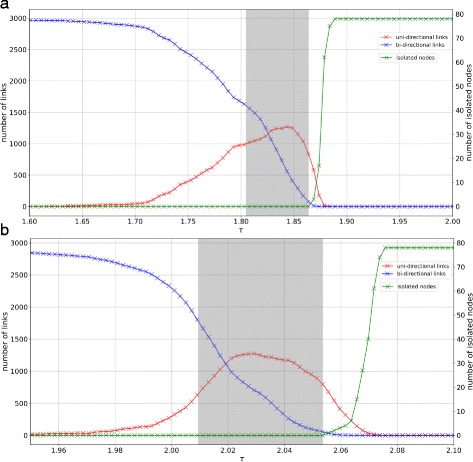
*Left* axis: change in link density (in absolute number of links) with respect to different values of *τ*. Our selection of *τ*_*min*_ corresponds to a relative link density of 0.7 (4204 links, counting each bi-directional link as two links). *Right* axis: number of isolated nodes. Our selection of *τ*_*max*_ is the highest possible *τ* for which there is still 1 weakly connected component (i.e. the network has no isolated nodes). The *grey* shaded areas indicate the resulting interval [*τ*_*min*_,*τ*_*max*_] for **a** *alpha2* and **b** *theta* band

The networks within [*τ*_*min*_,*τ*_*max*_] are all connected, but sparse enough to resemble complex structures. At *τ*_*max*_ itself, the link density is 0.168 for *alpha2* and 0.152 for *theta*, whereas the 30%-percentile of *τ*_*min*_ corresponds to networks with a link density of 0.7. This allows to cover a large variety of different networks in [*τ*_*min*_,*τ*_*max*_], each representing a different perspective on the underlying data. For example, we observe that the assortativity (Noldus and Van Mieghem 2015) for *theta* frequency band data ranges from −0.351 to −0.062 and that the ratio between uni-directional and bi-directional links is changing as well. Table 1 contains the exact values of *τ*_*min*_ and *τ*_*max*_ together with some properties of networks at the interval endpoints. Figure 3 shows how the number of links is changing for various sampled values of *τ*, including the interval.

**Table 1:** Network properties of *G*(*τ*) for *τ* at the endpoints of the interval [*τ*_*min*_,*τ*_*max*_]

	*alpha2*		*theta*	
	*G*(*τ*_*min*_)	*G*(*τ*_*max*_)	*G*(*τ*_*min*_)	*G*(*τ*_*max*_)
#uni-directional links	1006	848	648	799
#bi-directional links	1601	81	1776	56
average degree	53.949	12.949	53.846	11.679
assortativity	-0.105	-0.129	-0.351	-0.062
link density	0.700	0.168	0.700	0.152

For *alpha2* we have [*τ*_*min*_,*τ*_*max*_]=[1.8050,1.8636] and for *theta* [*τ*_*min*_,*τ*_*max*_]=[2.0095,2.0535]

## Information flow motifs

### Motif search

**Fig. 4:**
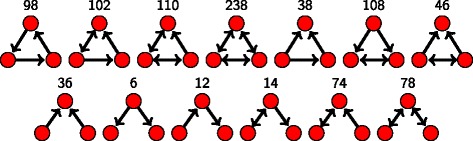
All 13 possible connected directed 3-motifs. The motif ID in binary represents the 3×3 adjacency matrix of the motif

**Fig. 5:**
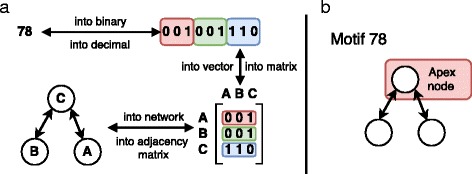
**a** Motif ids explained by motif 78 as an example. The decimal representation 78 encodes the binary adjacency matrix of the motif. **b** The central node of motif 78 is known as apex node

Our motif search is performed with the *mfinder* software version 1.2 (Kashtan et al. 2002). For the current study, our main focus is on the 13 different 3-motifs as shown in Fig. 4. Each motif is identified by a number whose binary representation translates to the adjacency matrix for the corresponding motif, consistent with the notation used by *mfinder*. Figure 5 gives an example of this conversion, using motif number 78 (the bi-directional 2-hop path).

For any given network *G* (to which we refer as “original network”), the *mfinder* program performs two tasks: first, it counts the frequency *J*_*G,M*_ of all motifs *M* in *G* and second, it generates a number of random networks with similar properties as the original network and determines the motif frequencies in each of them as well. For every original network, *mfinder* generates 1000 random networks using the switching algorithm described in Maslov and Sneppen (2002) with 100 switches. We use the default parameters for *mfinder*, which preserve the degree sequence of the original network and the number of bi-directional links.

The random networks serve as a null model to determine which motifs are *overexpressed* in the original network. More precisely, we adopt the criteria given in the supplemental material of Milo et al. (2002). These criteria are:

i) The probability that a motif in a random network occurs more or an equal amount of times as in the original network is smaller than 0.01.
ii) The motif appears in the original network at least 4 times with a distinct set of nodes.
iii) The ratio between the motif frequency of the original network and the average number of occurrences of the motif in the random networks is at least 1.1.

Given the mean *μ*(*J*_*rand,M*_) and the standard deviation *σ*(*J*_*rand,M*_) of the motif frequency in the random networks, the magnitude of overexpression of motif *M* in *G* is given by its *z*-score

3 $$ z_{G,M} = \frac{J_{G,M} - \mu(J_{rand,M})}{\sigma(J_{rand,M})}.  $$

A motif which is not overexpressed may still occur quite frequently in the original network, though it arises at a similar frequency by a random link rewiring process. Thus, it can be argued that overexpressed motifs carry some functional importance for the underlying system since they do not arise merely by chance.

### Overexpressed motifs in functional brain networks

**Fig. 6:**
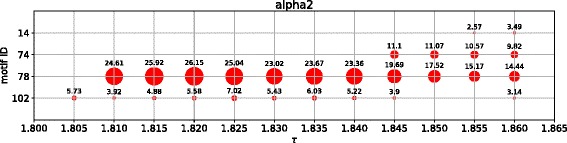
Overexpressed 3-motifs for *alpha2* band data in the interval [*τ*_*min*_,*τ*_*max*_]. The area of the circles scales with the *z*-scores. The numerical value of the *z*-scores is plotted on *top* of each *circle* for better comparison. Note that motif 78 has consistently the highest *z*-score

**Fig. 7:**
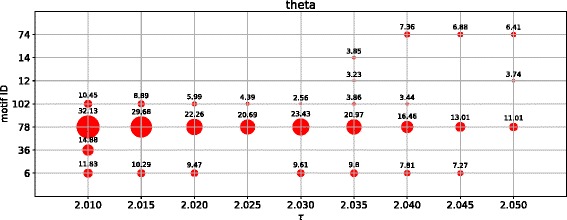
Overexpressed 3-motifs for *theta* band data in the interval [*τ*_*min*_,*τ*_*max*_]. The area of the circles scales with the *z*-score. The numerical value of the *z*-score is plotted on top of each circle for better comparison. Note that motif 78 has consistently the highest *z*-score

We sample the interval [*τ*_*min*_, *τ*_*max*_] with a step-size of *Δ*=0.005, for both *alpha2* and *theta* band data. For each sampled threshold *τ*, we construct *G*(*τ*) and regard *G*(*τ*) as the original network for *mfinder* in order to determine all overexpressed motifs. Figure 6 shows the overexpressed motifs for *alpha2* and Fig. 7 for *theta* band data together with the corresponding *z*-scores.

We observe that motif overexpression depends on the chosen threshold *τ*. For example, in the *alpha2* band motif 74 and motif 14 were only detected in very sparse networks close to the connectivity threshold *τ*_*max*_ (Fig. 6). Moreover, there are gaps at certain ranges of *τ* in which a motif does no longer fulfill all overexpression criteria, e.g. motif 102 at *τ*=1.85 and *τ*=1.855 for *alpha2* or motif 6 at *τ*=2.025 and *τ*=2.050 for *theta*.

From all overexpressed motifs, motif 78 stands out for the following reasons: Firstly, motif 78 is overexpressed in both, *alpha2* and *theta*, for a large part of the interval [*τ*_*min*_,*τ*_*max*_] without gaps between our sample points. Secondly, the *z*-scores for this motif are always higher than the *z*-scores of any other overexpressed motif for the corresponding thresholds. Hence, we select motif 78 as our motif *M* for the motif-based clustering in the “Motif-based clustering of functional brain networks” section.

### Apex-ratio and overlap with hubs

**Fig. 8:**
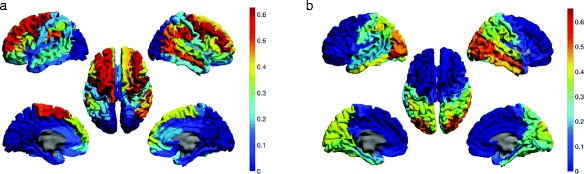
Average apex-ratio mapped to the template brain. The average was taken over equally distributed sample points of **a** [*τ*_*min*_,*τ*_*max*_]=[1.8050,1.8636] for *alpha2* and **b** [*τ*_*min*_,*τ*_*max*_]=[2.0095,2.0535] for *theta*. The step-size for sampling was *Δ*=0.005. The nodes with the highest apex-ratio in the *theta* band are found in posterior brain regions, where for *alpha2* the apex-ratio is lowest

Motif 78 encodes a pattern in which one central node is bi-directionally linked with two otherwise disconnected nodes. The node at this central position of motif 78 is known as *apex* and has been shown to be related to brain dynamics in previous studies (Harriger et al. 2012; Vicente et al. 2008; Gollo and Breakspear 2014; Gollo et al. 2014). The apex-ratio of a node is the ratio between the node occupying the apex-position (see Fig. 5b) divided by its total participation in instances of the complete motif 78. For example, an apex-ratio of 1 corresponds to a node that is always at the apex-position of motif 78, and never at a different position. Figure 8 shows a mapping of the average apex-ratio to the template brain for both frequency bands. The average was taken over equally distributed sample points, taken from the corresponding [*τ*_*min*_,*τ*_*max*_] with a step-size of *Δ*=0.005.

**Fig. 9:**
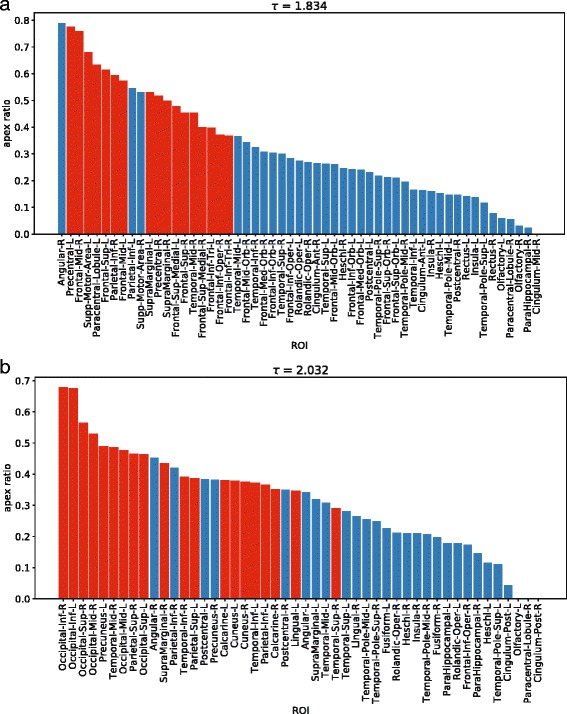
ROIs sorted in decreasing order by their apex-ratio. *Red bars* mark high-degree nodes, i.e. nodes with a degree higher than the average degree plus one standard deviation. **a** *alpha2* band for *τ*=1.834, **b** *theta* band for *τ*=2.032

Following the study by Sporns et al. (2007) conceptually, we are interested in the relation between the apex-ratio of a node and its degree. A node is a *high-degree node*, if its degree (number of incoming + outgoing links) is at least as large as the average degree of the network plus one standard deviation. Figure 9 shows that most of the nodes with the highest apex-ratio are also high-degree nodes in both the *alpha2* and *theta* band for *τ* fixed to $\frac 12(\tau _{min} + \tau _{max})$. While the apex-ratio and the number of high-degree nodes change with *τ*, we observe (not shown) a similar relation for different values of *τ* as well. More specifically, when considering the sample points between *τ*_*min*_ and *τ*_*max*_ described in the previous paragraph, the Pearson correlation coefficient between the apex-ratio and the degree for all nodes with a positive apex-ratio lies within [0.53,0.86] for *alpha2* and within [0.55,0.95] for *theta*.

## Motif-based clustering

Benson et al. (2016) developed a clustering algorithm that partitions a network *G* based on a motif *M*. The main idea of their algorithm is to construct clusters by “cutting” through the minimum possible number of motif instances, while maintaining a high density of motif instances within each of the clusters. In this section, we summarize only the basic concepts (including the algorithm) necessary to understand how the clustering of the networks was achieved. Details about the performance, complexity and additional applications can be found in the supplemental material of Benson et al. (2016) together with a comprehensive analysis of the algorithm.

### Motif adjacency matrices

Let *G* be a directed network with a set of nodes ${\mathcal {N}} = \{1, 2, \ldots, N\}$. Two motif instances are called *node-disjoint* if their set of nodes are not identical, i.e. they have at least one node not in common. For each pair of nodes *i*,*j* let *w*_*ij*_ be the number of node-disjoint motif instances in which *i* and *j* participate together. Then, the *N*×*N* symmetric matrix *W*_*M*_ with elements *w*_*ij*_ is called the *motif adjacency matrix*. The elements *d*_*ij*_ of the *motif diagonal degree matrix* *D*_*M*_ are given by

$$d_{ii} = \sum\limits_{j = 1}^{N}w_{ij} $$

and the *motif Laplacian* by

$$L_{M} = D_{M} - W_{M}. $$

**Fig. 10:**
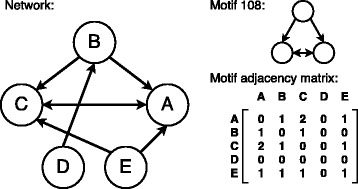
Example for the construction of a motif adjacency matrix based on the motif 108. In this example network, motif 108 can be found twice: 1. nodes {A, B, C}, 2. nodes {A, C, E}. Each instance of motif 108 is only counted once per set of nodes (node-disjoint motifs). The outgoing link from node D is still in the network, but is not part of any motif instance 108. The node-pair {A,C} is part of two different (node-disjoint) instances of motif 108, which is why there is a value of 2 at the corresponding cells of the motif adjacency matrix

The clustering algorithm uses the eigenvector belonging to the second smallest eigenvalue of the *normalized motif Laplacian*, which is defined as

$$\mathcal{L}_{M} = I - D_{M}^{-\frac{1}{2}} W_{M} D_{M}^{-\frac{1}{2}} $$

where *I* denotes the identity matrix. For a graph *G*(*τ*) based on a threshold *τ* the corresponding motif adjacency matrix is denoted by *W*_*M*_(*τ*). Figure 10 illustrates the construction of a motif adjacency matrix.

### Motif conductance

Given the motif adjacency matrix *W*_*M*_ of a network *G*, and a partition of the nodes $N = |\mathcal {N}|$ into two disjoint subsets $\mathcal {N}_{1}$ and $\mathcal {N}_{2} = \mathcal {N} \backslash \mathcal {N}_{1}$, we define the motif conductance $\phi _{G}({\mathcal {N}}_{1}, {\mathcal {N}}_{2})$ of that partition as

$$\phi_{G}(\mathcal{N}_{1}, \mathcal{N}_{2}) = \frac{\text{cut}_{G}\left(\mathcal{N}_{1},\mathcal{N}_{2}\right)}{\min\left\{ \text{vol}_{G}(\mathcal{N}_{1}), \text{vol}_{G}(\mathcal{N}_{2}) \right\} } $$

with

$$\text{cut}_{G}(\mathcal{N}_{1},\mathcal{N}_{2}) = \sum\limits_{i \in \mathcal{N}_{1},j \in \mathcal{N}_{2}} w_{ij} $$

and for *a*=1,2

$$\text{vol}_{G}(\mathcal{N}_{a}) = \sum\limits_{i \in \mathcal{N}_{a}}\sum\limits_{j}^{N} w_{ij} = \sum\limits_{i \in \mathcal{N}_{a}} d_{ii}. $$

Thus, the motif conductance $\phi _{G}(\mathcal {N}_{1}, \mathcal {N}_{2})$ equals the ratio between the number of motif-instances cut by the partition $\{\mathcal {N}_{1}, \mathcal {N}_{2}\}$ and the lowest number of preserved motif-instances in one of the two partitions.

### Motif-based clustering algorithm

A low conductance is often a desirable quality for a network clustering (Emmons et al. 2016). However, finding the minimum conductance of a network is a well-known $\mathcal {NP}$-complete problem (Garey and Johnson 2002) which directly translates to the complexity of finding the minimum motif conductance $\phi _{G}^{*}$. Benson et al. (2016) present a polynomial-time algorithm that finds a nearly optimal partition $\{ \mathcal {N}_{1}, \mathcal {N}_{2}\}$ with motif conductance

$$\phi_{G}(\mathcal{N}_{1}, \mathcal{N}_{2}) \leq 4 \sqrt{\phi_{G}^{*}} $$

for 3-motifs. In practice, the runtime is largely dominated by the computation of the motif adjacency matrix, which is still efficient for the motifs of size three that we consider for this work.

The algorithm from Benson et al. (2016) is a generalization of the classical spectral clustering algorithm (Van Mieghem 2011; Von Luxburg 2007), which makes use of the Laplacian matrix of a network. The eigenvector corresponding to the second smallest eigenvalue of this matrix is known as Fiedler’s vector (Fiedler 1973) and by ordering its elements, a node partition of a low (link-based) conductance can be devised.

The main steps of the algorithm from Benson et al. (2016) consist of computing the motif adjacency matrix *W*_*M*_ from which the normalized motif Laplacian $\mathcal {L}_{M}$ is constructed and the second smallest eigenvalue is computed. Afterwards, the corresponding eigenvector is used to create a partition $\{\mathcal {N}_{1}, \mathcal {N}_{2}\}$ according to the smallest motif conductance. Motif conductance is not defined for nodes that do not participate in any instance of the motif *M* and thus are not considered to be part of neither $\mathcal {N}_{1}$ nor $\mathcal {N}_{2}$. We show them as a separate third group of nodes.

The complete algorithm is listed as Algorithm 1 in pseudocode. We implemented the algorithm in Python (using NumPy and NetworkX) and double-checked our results with the implementation available on the SNAP-platform (Leskovec and Sosič R 2016).


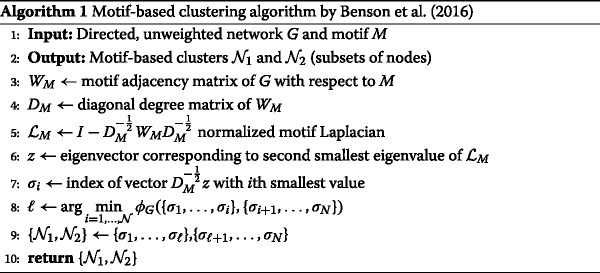


### Motif-based clustering of functional brain networks

The first step to apply the motif-based clustering to the brain is to fix a motif *M*. In the “Overexpressed motifs in functional brain networks” section, we identified motif 78 to be of high importance: it is prominent in both, the *alpha2* and *theta* band and provides continuously the highest *z*-score of all motifs, which designates it as the strongest candidate. Moreover, motif 78 is most robust against changes in *τ* as it was overexpressed at almost all sample points taken within [*τ*_*min*_,*τ*_*max*_]. However, it is not obvious, which of these sample points would result in the best possible network representation to create a meaningful clustering. To circumvent the selection of a fixed single threshold, we define a set of different thresholds T, each of them related to a different network and thus to different motif adjacency matrices. This is similar to the analysis done for Fig. 6, where we sampled [*τ*_*min*_,*τ*_*max*_] with a step-size of *Δ*=0.005, resulting in a set

$$\mathrm{T}^{*} = \left\{ \tau_{min} + k \cdot \Delta ~|~ k = 1, \ldots, 12 \right\}. $$

While this set is sufficient to get an idea about the impact of a changing *τ* on motif counts and makes for some compelling visualizations, equally distributed sample points result in a bias, since the change in the networks (i.e. their numbers of links) does not scale linearly with *τ* as shown in Fig. 3.

To avoid this bias, we pick the sample points T such that between each two consecutive sample points the corresponding networks change by the same amount. The smallest amount of change between two networks is the existence (or absence) of a single link. If we begin with the network *G*(*τ*=*τ*_*min*_) and slowly increase *τ* by *ε* until *G*(*τ*) and *G*(*τ*+*ε*) differ by exactly one link, we add *τ*+*ε* to our set T of sample points and continue this procedure until we eliminate the next link and so on. Thus, T consists of all thresholds *τ* within [*τ*_*min*_,*τ*_*max*_] at which the corresponding networks change by one link^2^, creating an unbiased sample of high resolution.

Summing the motif adjacency matrices over all networks generated by the elements in T results in an *aggregated motif adjacency matrix*

4 $$ W_{M_{agg}} = \sum\limits_{\tau \in \mathrm{T}} W_{M}(\tau)   $$

for each frequency band. Applying the motif-based clustering algorithm to the aggregated motif adjacency matrix given by Eq. (4) constructs a partition that takes the structure of different networks into account. Motifs consisting of strong links (i.e. with weights close to *τ*_*max*_) will be part of many of these networks, giving them more importance when searching for a partition of low motif conductance. In contrast, motifs with weak links (weights close to *τ*_*min*_) receive less consideration accordingly.

Although the aggregation avoids to base the complete analysis on a single fixed threshold, it introduces another difficult choice: the sample interval [*τ*_*min*_,*τ*_*max*_]. Our reasoning to set *τ*_*min*_ to the 30th-percentile of the PTE-distribution and *τ*_*max*_ to the weak connectivity threshold has been discussed already in the “Network construction” section. To add to this reasoning, we want to point out that in general, a small change to the endpoints from [*τ*_*min*_,*τ*_*max*_] will only result in small changes to aggregated clusterings, while a small change to a clustering based on a single threshold is comparably more sensitive. Ultimately, setting the interval [*τ*_*min*_,*τ*_*max*_] must, to some extent, remain a matter of *preference*, as it reflects which of the measurements (PTE values) are expected to be meaningful.

**Fig. 11:**
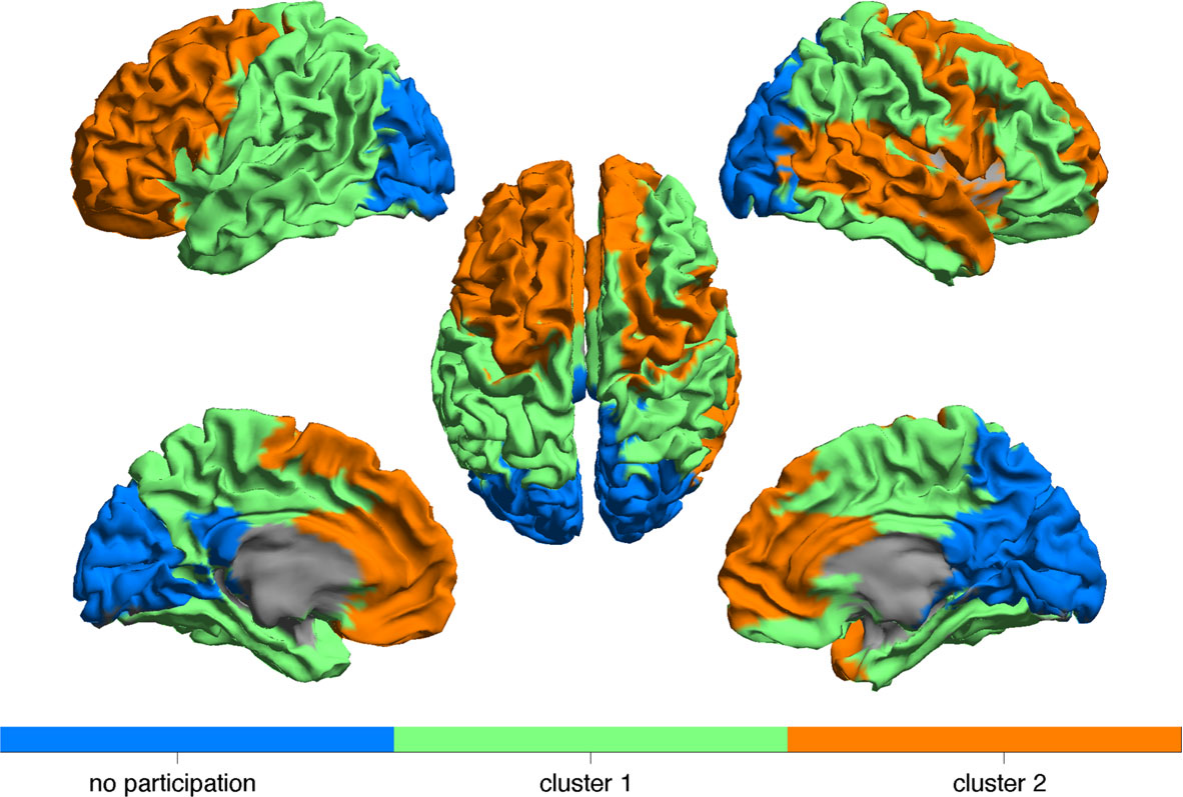
Partition of brain networks into two clusters of nodes based on motif 78 for the *alpha2* band. 15 out of 78 nodes did not participate in any motif 78 instance and are shown as a separate third cluster

**Fig. 12:**
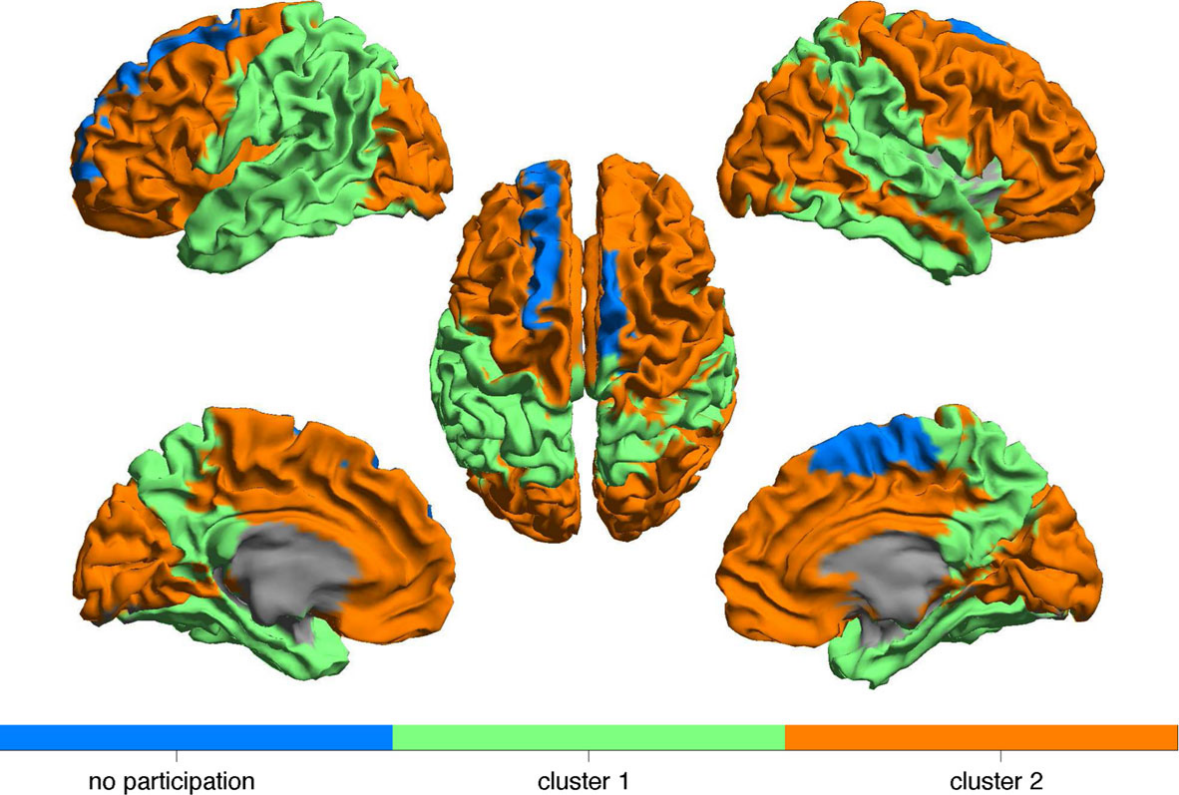
Partition of brain networks into two clusters of nodes based on motif 78 for the *theta* band. 2 out of 78 nodes did not participate in any motif 78 instance and are shown as a separate third cluster

The results of the partition of the brain into 2 clusters are shown in Fig. 11 for the *alpha2* band data and in Fig. 12 for the *theta* band data, based on our preference for [*τ*_*min*_,*τ*_*max*_].

## Discussion

### Overexpression of motif 78

Concerning network motifs, we observed an overexpression of motif 78 in line with our previous study (Meier et al. 2016). Two other motifs, 14 and 74, which can be regarded as degenerated forms of motif 78 missing one uni-directional link, have also been identified as overexpressed in both of our studies. Due to the overview over a range of thresholds in the current study, we can explain the origins of the overexpression of these related motifs: Since motifs 14 and 74 are only overexpressed for higher thresholds *τ* and, thus, only for sparser networks, their appearance seems to be a direct consequence of the applied threshold removing the weakest link in motif 78. Thus, motifs 14 and 74 are most likely consequences of the applied threshold not representing new triangular relations but supporting the overall dominance of motif 78.

The overexpression of motif 78 is also in line with previous research stating the same result for the structural brain networks of the macaque and the cat (Sporns and Kötter 2004). Gollo et al. (2015) applied neural mass models on the macaque connectome and identified motif 78 as an important motif for the dynamic core of the brain network. Furthermore, a recent study by Wei et al. (2017) singled out motif 78 as an important motif for the information transfer in functional brain networks. In particular, a node at the apex position of motif 78 acts as a *bridge* for the information flow between its neighbors and the overexpression of motif 78 could represent the basic principle of segregation and integration at the macroscopic level of brain regions (Sporns and Kötter 2004). The principle of segregation and integration originates from neuronal dynamics where signals from spatially segregated neurons are integrated with each other into one coherent signal (Sporns et al. 2004; Tononi et al. 1998; Zhigulin 2004). Further, Honey et al. (2007) showed that the participation of a node in motif 78 has a high correlation with being a hub of the network. The overexpression of motif 78 together with its close relation to hubs confirms previous findings identifying hubs as drivers for the integration of information flow (van den Heuvel et al. 2012; Gollo et al. 2015; Sporns et al. 2007). In addition, the overexpression of motif 78 in both frequency bands, *alpha2* and *theta*, strengthens the claim even further that motif 78 is a general building block of effective connectivity networks and therefore an important feature for the information flow in brain networks.

We showed that the hubs of the effective connectivity network often take on the apex position of motif 78. This hub-apex relation has previously been shown by (Sporns et al. 2007) for structural brain networks. We now extended this finding to the effective connectivity networks, identifying another shared feature of brain structure and function. The effective connectivity hubs seem to be located more in the front for the *alpha2* band and in posterior regions for the *theta* frequency band (Fig. 8). Considering these opposite locations together with the opposite directions of information flow that have been discovered by Hillebrand et al. (2016), these effective connectivity hubs seem to be the targets of the global information flow. Thus, one could argue that their target position in the global information flow patterns makes these hubs ’slaves’ of the information flow, which is line with a previous study by (Gollo et al. 2015). These findings support earlier studies by (Moon et al. 2015) and (Meier et al. 2017), which showed that hubs play an important role for the global network dynamics, and extend them from the structural to the functional domain.

### Clusters of the functional brain network

When analyzing the global intertwined organization of motif 78, we identified spatially coherent clusters in both frequency bands. Overall, the motif-based clustering algorithm split the brain in three major parts, the frontal lobe, the occipital lobe and the rest corresponding to a joint cluster of temporal and parietal lobe. Without including any spatial information in the construction of the directed networks or any restriction on locations for the performed clustering, we were able to recognize this well-known global spatial organization of the human brain in our obtained clusters.

As a commonality between the *alpha2* and *theta* band, the frontal regions seem to be nearly consistently together in one cluster. Moreover, in alignment with the recent study of Hillebrand et al. (2016) we also observe differences in the global patterns between high and low frequency bands. Whereas in the *theta* band, the posterior regions belong together with the frontal lobe in one cluster and thus participate in motif 78 together with the frontal lobe, the posterior regions in the *alpha2* band do not participate in motif 78. For the *theta* band, the frontal and the occipital lobe apparently share many interactions in the form of motif 78 because the clustering algorithm does not split them. This strong higher-order interaction between posterior and frontal brain regions could relate to the previously described global pattern of information flow between frontal and posterior regions in the *theta* band (Hillebrand et al. 2016; Dauwan et al. 2016).

The non-participating regions in the *alpha2* band consist mainly of strong hubs in posterior brain regions, which in our constructed networks have no in-degree but a significant out-degree. These nodes cannot participate in any instance of motif 78 as they would need at least one incoming link. Thus, the previously described pattern of information flow from the posterior to the frontal regions in the *alpha2* band is more likely based on the strong sending links, and less on this particular motif. However, the high density of motif 78 in the frontal regions might still play a role for the integration of the received signals from the posterior regions.

### Differences to previous study

We simplified the construction of directed networks in comparison with our previous study (Meier et al. 2016). In the earlier work, we computed the directed PTE (dPTE) value defined as

5 $$ \text{dPTE}_{XY}=\frac{\text{PTE}_{XY}}{\text{PTE}_{XY}+\text{PTE}_{YX}}  $$

for each direction and extracted the links with significantly high or low dPTE values. Thereby, we focused on the highly asymmetric pairwise relations representing strongly sending (dPTE>0.5) or strongly receiving nodes (dPTE<0.5), but discarded balanced nodes with a dPTE≈0.5. The dPTE is unable to distinguish whether both nodes are (at the same time) strong senders and receivers or are both weak senders and receivers. However, applying the PTE directly allows us to include those balanced nodes into our analysis, if they have strong enough connections (i.e. both directions have a PTE value greater than *τ*). Moreover, in contrast to our previous study (Meier et al. 2016), we did not fix a single threshold but analyzed how the motif counts and the corresponding results depend on the threshold. The clusters we find are based on a complete interval of thresholds and the remaining results on different sample points within this interval.

## Conclusions

The motif search for different frequency bands resulted in the dominant overexpression of motif 78 in networks generated over a wide range of thresholds. This motif, which was also observed in previous studies, seems to represent a general building block for the information flow in functional brain networks resembling the organizational principle of segregation and integration. The motif-based clustering revealed the higher-order organization of effective connectivity on a global scale. The differences between higher and lower frequency bands could be traced back to the interaction pattern between the posterior regions and the frontal regions. In the *theta* band, the frontal regions participated in many instances of motif 78 together with the posterior regions, pointing towards a strong integration of information flow between those spatially segregated areas. In the *alpha2* band, the posterior regions are no longer part of any cluster as they miss necessary bi-directional links to participate in motif 78, although the segregation between the frontal regions and the remainder of the brain is still observable. Further investigation into other over-expressed motifs may shed more light on similar principles of information flow in the brain.

## Endnotes

^1^ The MEG data were recorded using a 306-channel whole-head MEG system (Elekta Neuromag Oy, Helsinki, Finland) during a no-task, eyes-closed condition for five consecutive minutes. A beamformer approach was adopted to project MEG data from sensor space to source space (Hillebrand et al. 2012) and the automated anatomical labelling (AAL) atlas was applied to obtain time series for 78 cortical regions of interest (ROIs) (Gong et al. 2009; Tzourio-Mazoyer et al. 2002). For each subject, we extracted the first 20 artefact-free epochs of 4096 samples (3.2768 s).

^2^ Note that the values in T are exactly the PTE values of the links that get removed by this procedure.

## Abbreviations

DMN: Default mode network
dPTE: Directed phase transfer entropy
MEG: magnetoencephalography
PTE: Phase transfer entropy
ROI: Region of interest
